# Phthalate induced testicular degeneration in mice: biochemical, histopathological and immunohistochemical evidence for the interplay of oxidative stress, caspase-3, and NF-κB signaling

**DOI:** 10.1038/s41598-026-63843-y

**Published:** 2026-07-29

**Authors:** Reda A. Ali, Heba E. Aboulqasem, Dalia Elzahraa F. Mostafa

**Affiliations:** Zoology and Entomology Department, Faculty of Science, Assiut University, Assiut, 71516 Egypt

**Keywords:** DBP, Testicular toxicity, LPO, GSH, DEHP, Histopathology, Biochemistry, Cell biology, Diseases, Medical research, Physiology

## Abstract

Phthalates, including dibutyl phthalate (DBP) and di(2-ethylhexyl) phthalate (DEHP), are widely used plasticizers associated with male reproductive toxicity. This study evaluated the dose- and temporal-dependent effects of DBP and DEHP (100, 200, and 400 mg/kg for 15 days) on testicular structure and function in immature Swiss albino mice at 36, 45, 50, and 70 days of age. Biochemical analyses revealed significant glutathione depletion accompanied by increased nitric oxide and lipid peroxidation levels, suggesting oxidative imbalance. Histological evaluation demonstrated severe dose-dependent testicular injury, particularly at 36–45 days, with the 400 mg/kg groups showing 100% damaged seminiferous tubules and marked reductions in Johnsen’s scores (3.5 ± 0.17 vs. 9.9 ± 0.10 in controls). Increased Caspase-3 and NF-κB expression suggested possible involvement of apoptotic and inflammatory responses. Longitudinal assessment from 50 to 70 days revealed a dose-dependent recovery pattern, with substantial restoration in lower-dose groups, whereas higher doses remained associated with persistent tubular damage at 70 days (47.0% in DBP and 58.8% in DEHP groups). These findings suggest that the severity of testicular injury and the extent of recovery are influenced by phthalate exposure dose. Therefore, further studies are required to fully understand the underlying molecular mechanisms.

## Introduction

Infertility is a widespread global health challenge, with the World Health Organization estimating that about one in six people (≈ 17.5%) experience infertility at some point in their lives. While the precise proportion attributable to male factors varies among regions, many studies suggested that male infertility contributes substantially to overall infertility rates^1^. This rising prevalence emphasizes the need to understand the underlying causes and develop effective strategies to mitigate their impact on reproductive health. Among the contributing factors, environmental endocrine disruptors (EEDs), particularly phthalates, have attracted significant attention due to their widespread presence and potential adverse effects^2^.

Phthalates are widely present in everyday life and are commonly incorporated to improve the flexibility, strength, and lifespan of various consumer products. They are particularly used in the manufacture of polyvinyl chloride (PVC) plastics, which are found in construction materials, medical equipment, food packaging, and children’s toys^3,4^. Beyond plastics, phthalates are also components of many personal care items, including cosmetics, perfumes, and shampoos, where they function as solvents and stabilizers^5^. Due to their non-covalent integration into products, phthalates can migrate into the environment through leaching, volatilization, or wear, leading to widespread contamination^6^. Among these chemicals, di(2-ethylhexyl) phthalate (DEHP) is the most commonly detected in environmental samples. Cumulative exposure from multiple sources can reach levels that may pose considerable health risks. Several phthalates, such as DEHP, dibutyl phthalate (DBP), di-isobutyl phthalate (DIBP), and butyl benzyl phthalate (BBP), are recognized for their reproductive toxicity and endocrine-disrupting effects. Although regulatory measures have been established globally, continuous monitoring is crucial to reduce the potential health hazards associated with long term exposure^7^.

Phthalate exposure has been frequently linked to reproductive health disturbances in both males and females^8,9^. Previous studies have shown that DEHP can impair sperm motility, disrupt sperm maturation, and reduce circulating testosterone levels^10^. Furthermore, phthalates may alter the size and function of male reproductive organs, such as the testes and prostate, thereby potentially increasing the risk of infertility^11^. Recent systematic evidence refers to phthalates as “silent saboteurs” of male fertility, reflecting their consistent association with impaired semen quality and disrupted spermatogenesis^12^.

At the mechanistic level, phthalates disrupt redox homeostasis by increasing reactive oxygen species (ROS) generation and depleting endogenous antioxidant defenses, thereby promoting lipid peroxidation and germ cell damage^13^. They are also associated with male reproductive toxicity through multiple interconnected mechanisms, including oxidative stress, endocrine disruption, inflammation, and apoptosis^14^. These molecular disruptions in animal models exposed to phthalates include dose dependent testicular damage characterized by degeneration of the seminiferous tubules and compromised spermatogenesis due to impaired blood testis barrier integrity^15^.

Although the effects of phthalates on oxidative stress, inflammation, and apoptosis are well documented, relatively few studies have simultaneously assessed biochemical, histopathological, and immunohistochemical alterations across different doses. Accordingly, this study was designed to investigate the effects of DBP and DEHP exposure on oxidative stress, antioxidant defense systems, histopathological changes, and apoptotic and inflammatory markers in testicular tissue. It is hypothesized that DBP and DEHP may induce dose-dependent testicular toxicity potentially involving oxidative stress, apoptosis, and inflammation. Importantly, evaluating these parameters across graded doses and different time points may help identify sensitive windows of testicular susceptibility and provide insight into which exposure levels are associated with recovery versus persistent testicular alterations.

## Results

### Biochemical statistical analysis

#### Glutathione (GSH) level

##### At the age of 36 days

**Fig. 1:**
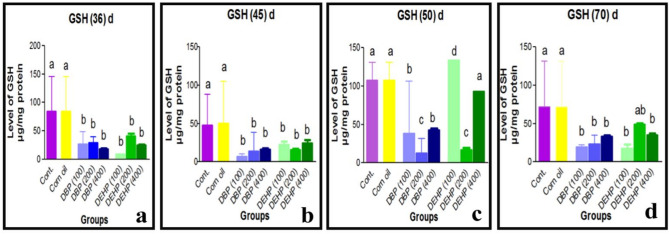
The levels of GSH (µg/mg protein) in treated groups at different ages, arranged in ascending order as follows: (**a**) 36 days, (**b**) 45 days, (**c**) 50 days and (**d**) 70 days. Different letters denote significant differences between groups (*P* < 0.05), whereas groups sharing the same letter are insignificantly different.

All treated groups exhibited a significant decline in GSH levels compared to control (*p* < 0.0001, F_7,16_ = 15.16, ƞp^2^ = 0.87; Fig. 1a). The reduction was consistent across DBP and DEHP groups, all of which were statistically comparable to each other but significantly different from the control.

##### At the age of 45 days

GSH levels were significantly depleted in all treated groups compared to control and corn oil groups (*p* = 0.0010, F_7,16_ = 6.452, ƞp^2^ = 0.738408; Fig. 1b). No significant variation was observed among the DBP or DEHP groups at this stage.

##### At the age of 50 days

Significant depletion was observed in DBP (100, 200, and 400 mg/kg) and DEHP (200 mg/kg) groups compared to the control (*p* < 0.0001, F_7,16_ = 51.71, ƞp² = 0.96; Fig. 1c). Notably, the lowest values were detected at the 200 mg/kg dose in both DBP and DEHP treatments. DEHP (100 mg/kg) showed a unique significant elevation, whereas DEHP (400 mg/kg) did not significantly differ from control.

##### At the age of 70 days

GSH levels were significantly reduced in all DBP treated groups and DEHP (100 and 400 mg/kg) compared to the control (*p* = 0.0001, F_7,16_ = 9.308, ƞp² = 0.80; Fig. 1d). DEHP (200 mg/kg) exhibited a level that was insignificantly different from control.

#### Nitric oxide (NO) level

##### At the age of 36 days

**Fig. 2:**
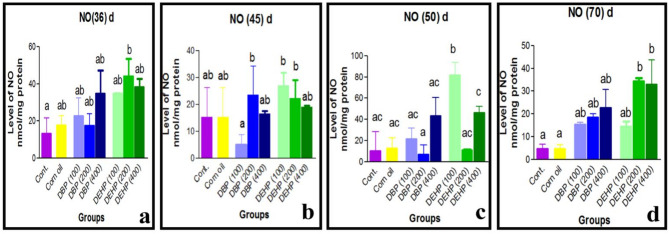
The levels of NO (nmol/mg protein) in treated groups at different ages, arranged in ascending order as follows: (**a**) 36 days, (**b**) 45 days, (**c**) 50 days and (**d**) 70 days. Different letters denote significant differences between groups (*P* < 0.05), whereas groups sharing the same letter are insignificantly different.

A significant elevation in NO levels was observed in the DEHP (200 mg/kg) group compared to the control (*p* = 0.0100, F_7,16_ = 4.024, ƞp² = 0.64; Fig. 2a). Meanwhile, all DBP doses and the remaining DEHP doses showed increases that were not statistically significant compared with control group.

##### At the age of 45 days

NO levels were significantly elevated in the DEHP (100 and 200 mg/kg) and DBP (200 mg/kg) groups compared to other treated groups (*p* = 0.0120, F_7,16_ = 3.857, ƞp² = 0.63; Fig. 2b), but the differences were insignificant compared to control. The highest elevation was observed in DEHP (100 mg/kg) group, while DBP (100 mg/kg) showed the lowest levels, which were significantly different from the elevated groups.

##### At the age of 50 days

DEHP (100 mg/kg) group exhibited a significant and prominent elevation compared to control. Followed by DEHP (400 mg/kg), which also showed a significant increase (*p* < 0.0001, F_7,16_ = 10.65, ƞp² = 0.82; Fig. 2c). However, all DBP treated groups and DEHP (200 mg/kg) remained statistically similar to control group.

##### At the age of 70 days

NO levels were significantly elevated in the DEHP (200 and 400 mg/kg) groups compared to control. (*p* = 0.0023, F_7,16_ = 5.514, ƞp² = 0.71; Fig. 2d). In contrast, all DBP treated groups and DEHP (100) showed increased levels compared to control, but the differences were statistically insignificant.

#### Lipid peroxidation (LPO) level

##### At the age of 36 days

**Fig. 3:**
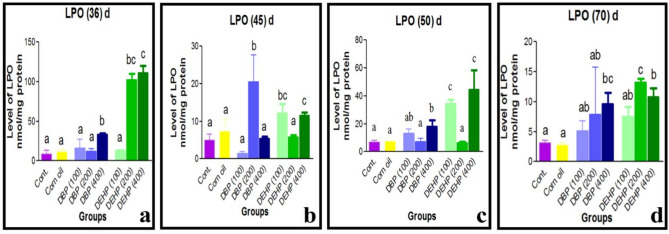
The levels of lipid peroxidation LPO (nmol/mg protein) in treated groups at different ages, arranged in ascending order as follows: (**a**) 36 days, (**b**) 45 days, (**c**) 50 days and (**d**) 70 days. Different letters denote significant differences between groups (*P* < 0.05), whereas groups sharing the same letter are insignificantly different.

LPO levels were dramatically and significantly elevated in DEHP (200 and 400 mg/kg) groups compared to control (*p* < 0.0001, F_7,16_ = 94.09, ƞp² = 0.98; Fig. 3a). In contrast, all DBP treated groups and DEHP (100 mg/kg) exhibited much lower levels, showing insignificant difference from control group.

##### At the age of 45 days

DBP (200 mg/kg) group showed the most significant elevation compared to control, followed by significant increases in DEHP (100 and 400 mg/kg) (*p* < 0.0001, F_7,16_ = 27.09, ƞp² = 0.92; Fig. 3b). In contrast, DBP (100 and 400 mg/kg) groups and the mid dose DEHP (200 mg/kg) group remained statistically comparable to control.

##### At the age of 50 days

LPO levels were significantly increased in the DBP (400 mg/kg) and DEHP (100 and 400 mg/kg) groups compared with the control group (*p* < 0.0001, F_7,16_ = 7.5, ηp² = 0.8; Fig. 3c), whereas no significant differences were observed in the DBP (100 and 200 mg/kg) or DEHP (200 mg/kg) groups.

##### At the age of 70 days

Levels were significantly elevated in DEHP treated groups, with the highest observed level in DEHP (200 mg/kg) followed by DEHP (100 and 400 mg/kg) (*p* = 0.0001, F_7,16_= 9.228, ƞp² = 0.80; Fig. 3d). In contrast, all DBP treated groups exhibited levels that were insignificantly different from control.

### Histopathological investigations

#### Masson’s trichrome stain

**Fig. 4:**
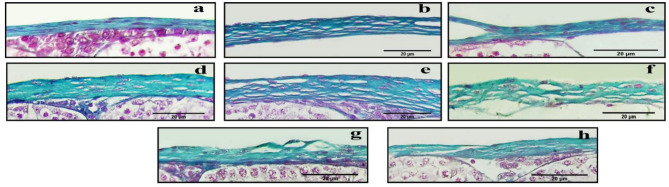
Representative photomicrographs showing the tunica albuginea of testicular sections at different ages: (**a**) Control; (**b**,** c**) B (400 mg/kg) at 36 days; (**d**) E (100 mg/kg) at 36 days; (**e**) E (200 mg/kg) at 36 days; (**f**) B (400 mg/kg) at 45 days; (**g**) E (100 mg/kg) at 45 days; and (**h**) E (200 mg/kg) at 45 days. ×400. (Masson’s trichrome).

At all examined ages (36–70), the tunica albuginea is a dense fibrous connective tissue capsule surrounding the testis (Fig. 4a). Its thickness and structure remained consistent across all ages, showing no appreciable morphological changes. In treated groups, the tunica albuginea showed subtle alterations compared to controls. These alterations included fibrotic changes (Fig. 4b-h), localized increases in nuclear density (Fig. 4c) and occasional disorganization of collagen fibers (Fig. 4e-g). Minor fragmentation of the fibrous matrix (Fig. 4h) were also observed.

#### Hematoxylin and Eosin (H&E) stain

##### At the age of 36 days

**Fig. 5:**
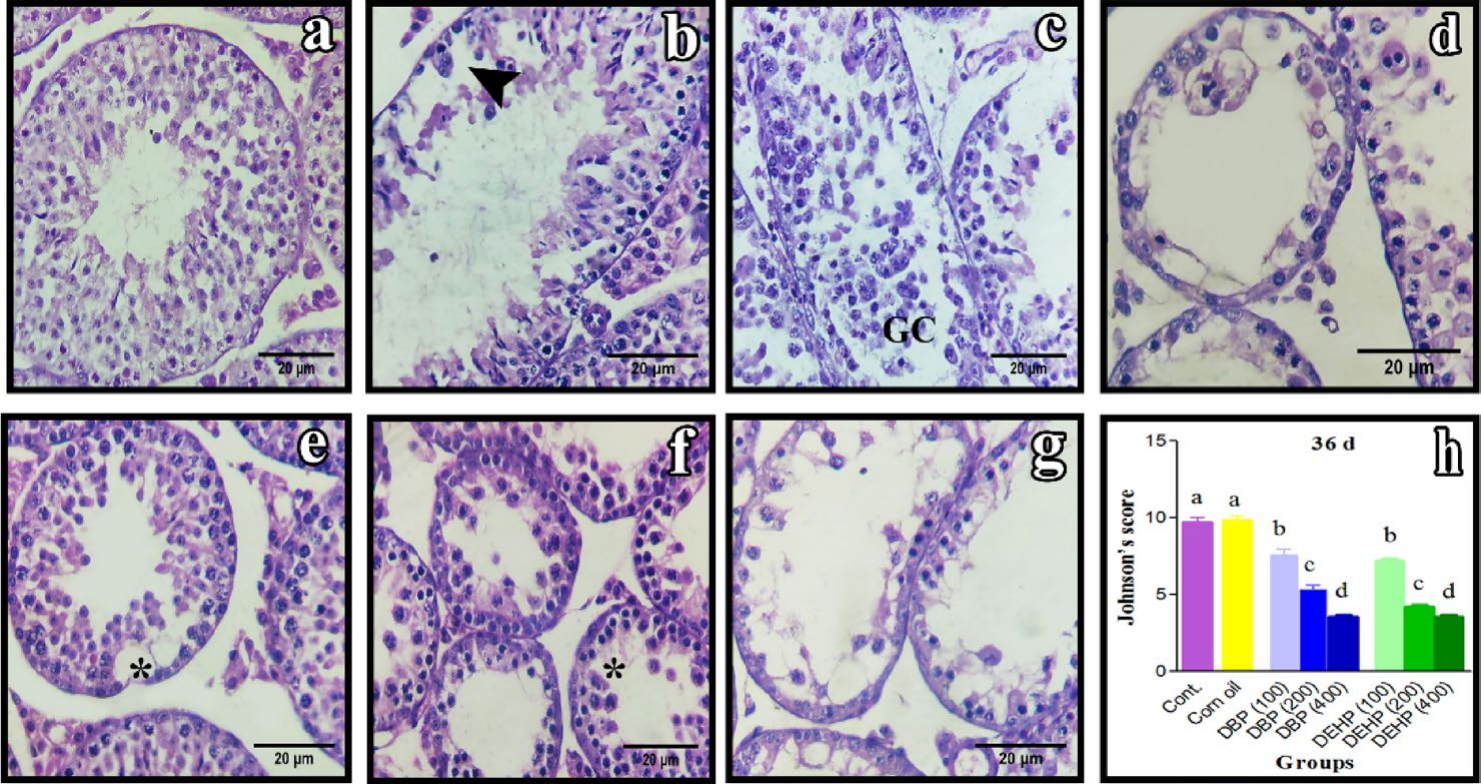
Representative photomicrographs of testicular sections at the age of 36 days stained with H&E. (**a**) Control: normal spermatogenesis with elongated spermatids and occasional spermatozoa in the tubular lumen. (**b**) DBP 100 mg/kg: focal loss of germ cells (head arrow). (**c**) DBP 200 mg/kg: disorganization of germ cells (GC). (**d**) DBP 400 mg/kg and (**g**) DEHP 400 mg/kg: germ cells largely absent leaving only spermatogonia. (**e**) DEHP 100 mg/kg and (**f**) DEHP 200 mg/kg: cytoplasmic vacuolization (star). ×400. (h) Johnsen’s score across experimental groups. Different letters denote significant differences between groups (*P* < 0.05), whereas groups sharing the same letter are insignificantly different.

In control, elongated spermatids and occasional spermatozoa were observed within the tubular lumen, indicating near completion of the first wave of spermatogenesis (Fig. 5a). In treated groups, variable testicular alterations were observed, including partial, focal loss of germ cells within affected seminiferous tubules (Fig. 5b) and disorganization of germ cells with luminal displacement (Fig. 5c). In the most severely affected samples, germ cells were largely absent, leaving only spermatogonia (Fig. 5d & g). Cytoplasmic vacuolization was also evident (Fig. 5e & f). These histopathological changes were supported by Johnson’s scoring system, which demonstrated a significant dose-dependent reduction in testicular integrity (Fig. 5h), with progressive deterioration in both DBP- and DEHP-treated groups. The mean Johnsen score decreased from 9.7 ± 0.15 (control) and 9.8 ± 0.13 (corn oil) to 7.6 ± 0.16, 5.3 ± 0.15, and 3.5 ± 0.17 in DBP groups, and 7.2 ± 0.13, 4.2 ± 0.13, and 3.5 ± 0.17 in DEHP groups (100, 200, and 400 mg/kg, respectively). Quantitative analysis of damaged seminiferous tubules confirmed a statistically significant increase in the percentage of damaged tubules in all treated groups compared with control (Fig. 9a). The values were 6.1% in the control group and 7.1% in the corn oil group, compared with 39.1%, 70.0%, and 100% in DBP-treated groups, and 25.0%, 62.5%, and 100% in DEHP-treated groups (100, 200, and 400 mg/kg, respectively).

##### At the age of 45 days

**Fig. 6:**
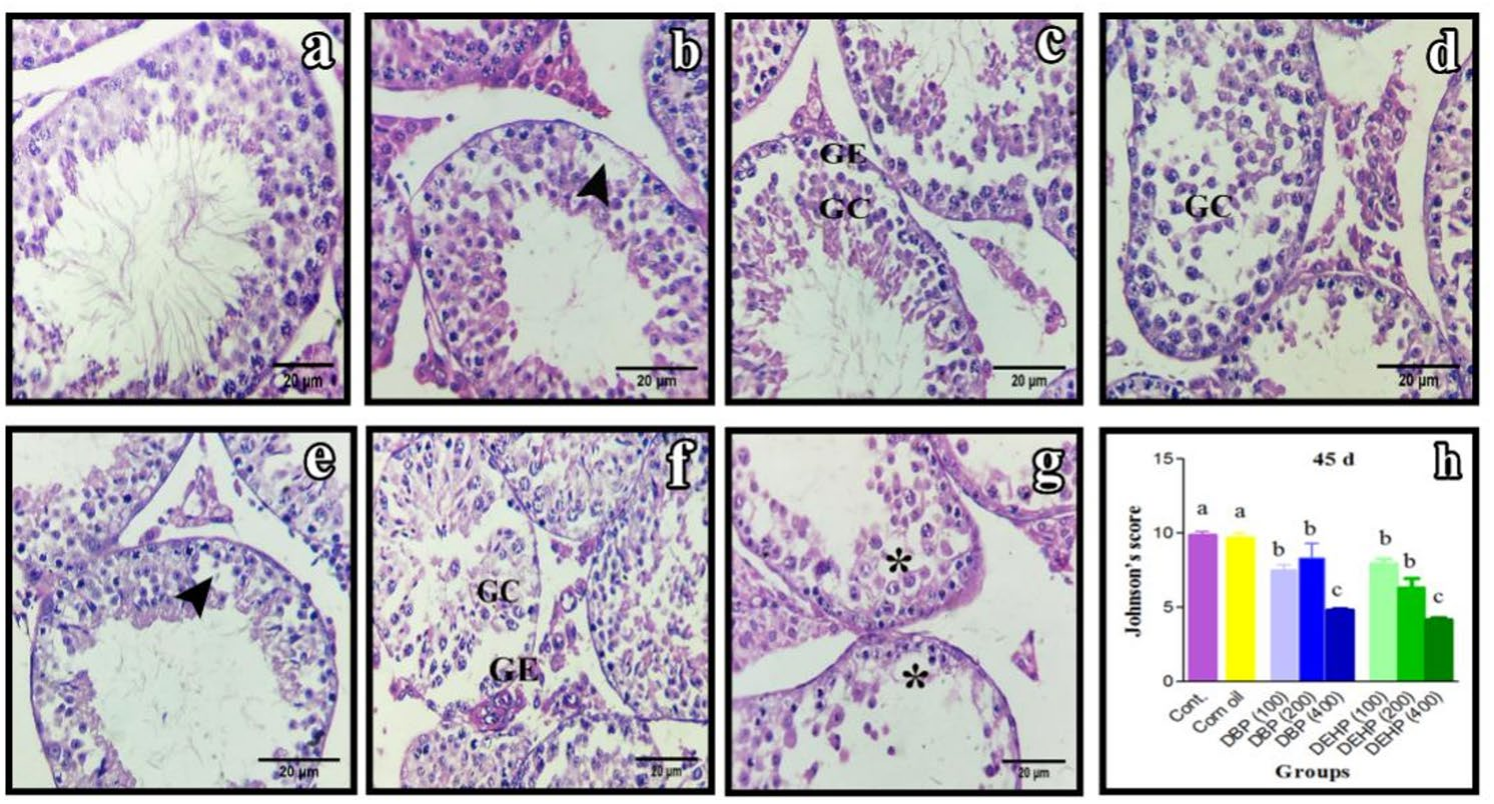
Representative photomicrographs of testicular sections at the age of 45 days stained with H&E. (**a**) Control: fully mature spermatogenesis. (**b**) DBP 100 mg/kg: focal loss of germ cells (head arrow). (**c**) DBP 200 mg/kg: germ cell (GC) detachment from the germinal epithelium (GE). (**d**) DBP 400 mg/kg: dispersed degenerating germ cells (GC). (**e**) DEHP 100 mg/kg: focal loss of germ cells (head arrow). (**f**) DEHP 200 mg/kg: germ cell (GC) detachment from the germinal epithelium (GE). (**g**) DEHP 400 mg/kg: marked vacuolization (star). ×400. (**h**) Johnsen’s score across experimental groups. Different letters denote significant differences between groups (*P* < 0.05), whereas groups sharing the same letter are insignificantly different.

Control seminiferous tubules displayed a fully mature spermatogenic pattern, with all germ cell stages represented and abundant spermatozoa filling the tubular lumen (Fig. 6a). In the treated groups, focal loss of germ cells was observed within affected seminiferous tubules (Fig. 6b & e). Some germ cells appeared dislodged from their basal positions, while the spermatogonial layer remained relatively intact (Fig. 6c & f). In samples exposed to higher doses, severe testicular injury was evident, characterized by dispersed degenerating germ cells (Fig. 6 d) and a marked increase in cytoplasmic vacuolization within the seminiferous tubules (Fig. 6 g). Quantitative analysis using Johnson’s scoring system confirmed a significant dose-dependent impairment in testicular structure (Fig. 6 h). The mean scores were 9.9 ± 0.10 and 9.7 ± 0.15 in control and corn oil groups, respectively. DBP-treated groups showed values of 7.5 ± 0.17, 8.3 ± 0.45, and 4.8 ± 0.13, while DEHP-treated groups exhibited scores of 7.9 ± 0.38, 6.3 ± 0.68, and 4.2 ± 0.13 (100, 200, and 400 mg/kg, respectively). Quantitative analysis of damaged seminiferous tubules further confirmed these findings (Fig. 9b). The percentage of damaged tubules remained significantly elevated in all treated groups, showing dose-dependent increases of 25.0%, 47.0%, and 73.3% in DBP-treated animals and 25.0%, 37.0%, and 92.0% in DEHP-treated animals (100, 200, and 400 mg/kg, respectively), compared with low baseline levels in the control and corn oil groups (4.0% and 11.1%, respectively).

##### At the age of 50 days

**Fig. 7:**
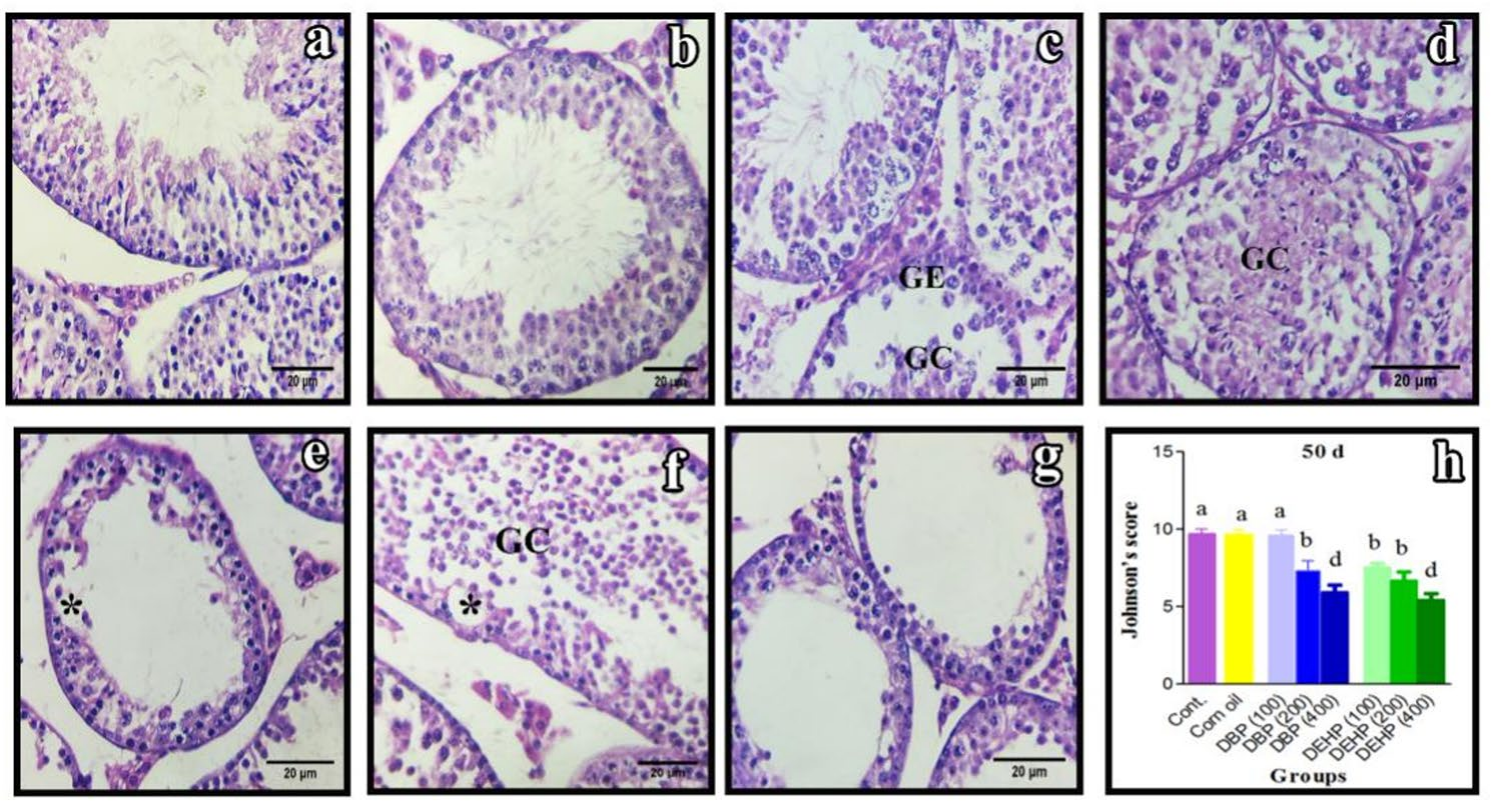
Representative photomicrographs of testicular sections at the age of 50 days stained with H&E. (**a**) Control: stable testicular architecture comparable to adult mice. (**b**) DBP 100 mg/kg: relatively preserved germ cell organization. (**c**) DBP 200 mg/kg: germ cell (GC) detachment from the germinal epithelium (GE). (**d**) DBP 400 mg/kg: dispersed germ cells (GC). (**e**) DEHP 100 mg/kg and (**f**) DEHP 200 mg/kg: marked cytoplasmic vacuolization (star). (**g**) DEHP 400 mg/kg: marked germ cell loss. ×400. (**h**) Johnsen’s score across experimental groups. Different letters denote significant differences between groups (*P* < 0.05), whereas groups sharing the same letter are insignificantly different.

In control, the testicular architecture appeared stable and comparable to adult mice, indicating sustained and efficient spermatogenesis (Fig. 7a). In treated groups, partial improvement was observed in the lower exposure groups, with most seminiferous tubules showing relatively preserved germ cell organization (Fig. 7b). In the intermediate exposure groups, some tubules exhibited structural damage, including detachment of germ cells from the germinal epithelium (Fig. 7c) and marked increase in vacuolization of germ cells within the seminiferous tubules (Fig. 7e & f). High exposure groups displayed severe alterations, with germ cells dispersed into the tubular lumen (Fig. 7 d & f) and widespread disruption of spermatogenic architecture (Fig. 7c & d). Marked germ cell loss was also evident (Fig. 7 g). Johnson’s scoring analysis demonstrated a persistent dose-dependent reduction in testicular integrity (Fig. 7 h). The mean scores were 9.7 ± 0.15 (control) and 9.6 ± 0.16 (corn oil). DBP-treated groups showed scores of 9.6 ± 0.16, 7.3 ± 0.30, and 5.9 ± 0.53, whereas DEHP-treated groups exhibited scores of 7.5 ± 0.31, 6.7 ± 0.60, and 5.4 ± 0.48 (100, 200, and 400 mg/kg, respectively). Consistent with these findings, quantitative analysis revealed a statistically significant dose-dependent increase in the percentage of damaged seminiferous tubules relative to the control (Fig. 9c). The percentages were 6.6% in the control group and 17.6% in the corn oil group, compared with 29.6%, 50.0%, and 46.4% in DBP-treated groups, and 38.0%, 47.4%, and 51.5% in DEHP-treated groups (100, 200, and 400 mg/kg, respectively).

##### At the age of 70 days

**Fig. 8:**
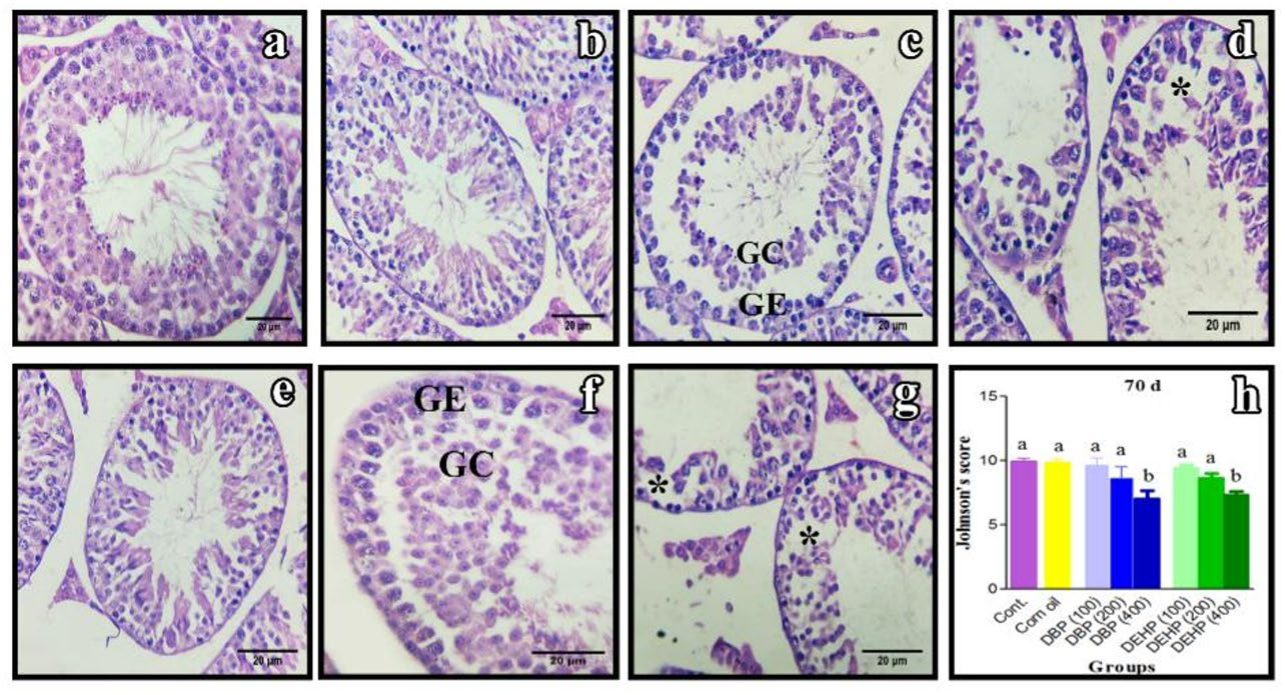
Representative photomicrographs of testicular sections at the age of 70 days stained with H&E. (**a**) Control: classic adult seminiferous tubule architecture. (**b**) DBP 100 mg/kg: partial tubule restoration. (**c**) DBP 200 mg/kg: germ cell (GC) detachment from the germinal epithelium (GE). (**d**) DBP 400 mg/kg and DEHP 400 mg/kg: focal vacuolization (star). (**e**) DEHP 100 mg/kg: partial tubule restoration. (**f**) DEHP 200 mg/kg: germ cell (GC) detachment from the germinal epithelium (GE). ×400. (h) Johnsen’s score across experimental groups. Different letters denote significant differences between groups (*P* < 0.05), whereas groups sharing the same letter are insignificantly different.

**Fig. 9:**
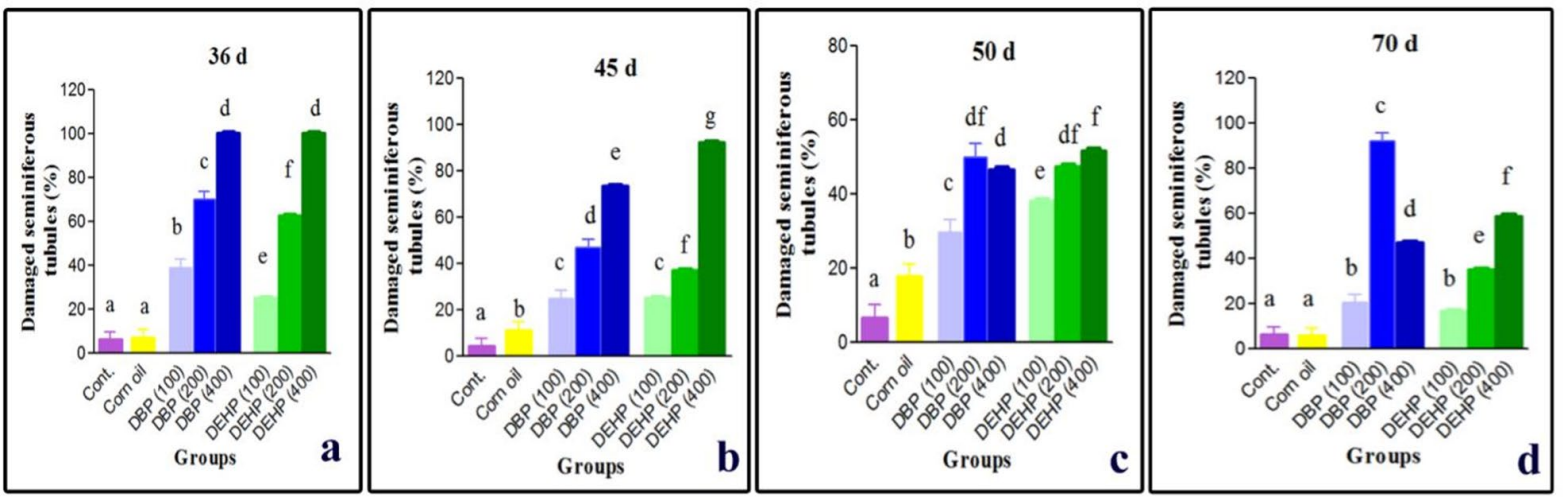
Percentage of degenerated seminiferous tubules in treated groups at different ages, arranged in ascending order as follows: (**a**) 36 days, (**b**) 45 days, (**c**) 50 days and (**d**) 70 days. Different letters denote significant differences between groups (*P* < 0.05), whereas groups sharing the same letter are insignificantly different.

Testes at 70 days exhibited classic adult seminiferous tubule architecture with continuous spermatogenic activity and densely packed spermatozoa within the lumen (Fig. 8a). Overall improvement was observed in the treated groups, particularly in the lower exposure groups, which closely resembled the control samples and exhibited restoration of seminiferous tubule architecture and spermatogenesis (Fig. 8b & e). In contrast, the higher exposure groups still displayed occasional detachment of germ cells from the germinal epithelium (Fig. 8c & f) and focal vacuolization (Fig. 8 d & g). Despite partial recovery, Johnson’s scoring system indicated persistent long-term effects of phthalate exposure (Fig. 8 h). Mean scores were 9.9 ± 0.10 (control) and 9.8 ± 0.13 (corn oil). DBP-treated groups showed values of 9.6 ± 0.27, 8.6 ± 0.40, and 7.0 ± 0.63, while DEHP-treated groups showed 9.4 ± 0.27, 8.6 ± 0.40, and 7.3 ± 0.30 (100, 200, and 400 mg/kg, respectively). Quantitative analysis of damaged seminiferous tubules further supported these findings (Fig. 9 d). A marked reduction in tubular damage was observed in the low-dose groups, consistent with partial restoration of spermatogenesis. The percentage of damaged tubules was 6.0% in the control group and 5.7% in the corn oil group, compared with 20.6%, 92.0%, and 47.0% in DBP-treated groups, and 16.6%, 35.0%, and 58.8% in DEHP-treated groups (100, 200, and 400 mg/kg, respectively).

### Immunohistochemical investigations

#### At 36 days of age

##### Caspase-3

**Fig. 10:**
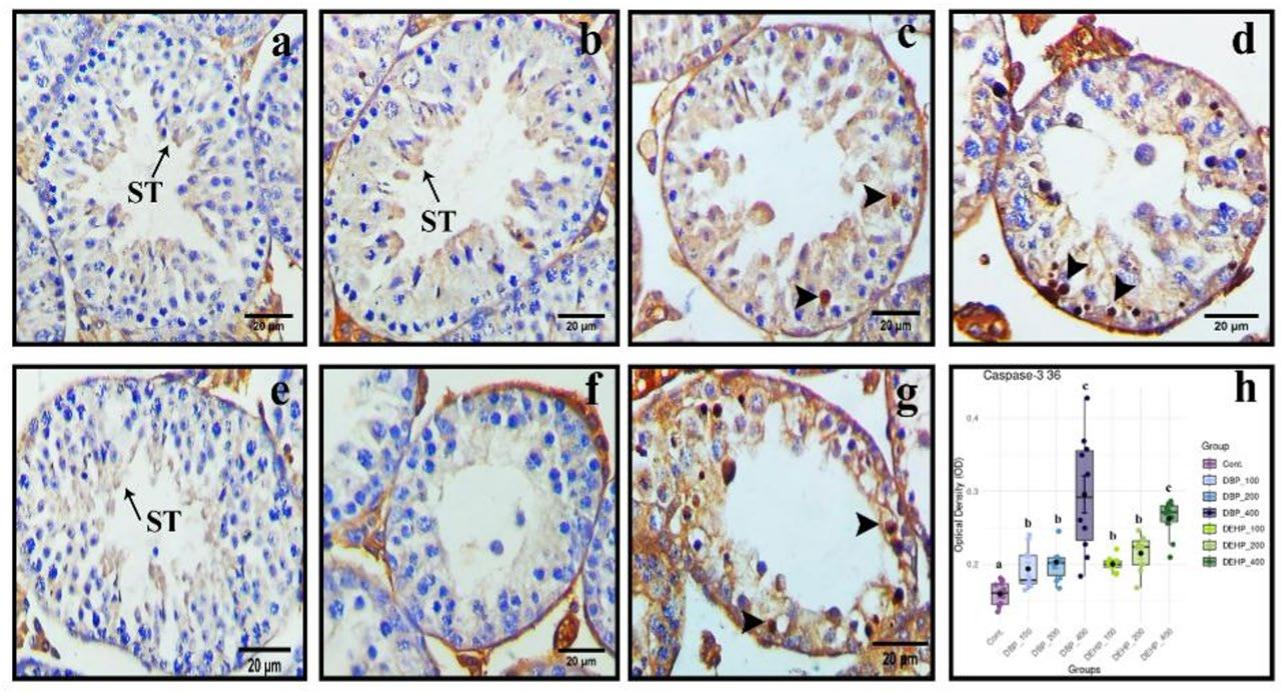
Immunohistochemical detection of caspase-3 in testicular tissue at age of 36 days. Representative micrographs: (**a**) Control, (**b**) DBP 100 mg/kg, (**c**) DBP 200 mg/kg, (**d**) DBP 400 mg/kg, (**e**) DEHP 100 mg/kg, (**f**) DEHP 200 mg/kg, (**g**) DEHP 400 mg/kg. Germ cells (arrowheads) & spermatids (arrows, ST). ×400. (h) Optical density (OD) measurements. Different letters denote significant differences between groups (*P* < 0.05), whereas groups sharing the same letter are insignificantly different.

Caspase-3 expression in the control and in the low dose groups was observed only in the cytoplasm of spermatids within the seminiferous tubules (Fig. 10a, b & e). In higher dose treated groups, expression was detected in the nuclei of some germ cells, with the strongest staining observed in the high doses of DBP (200 & 400 mg/kg) (Fig. 10c & d) and the highest dose of DEHP (400 mg/kg) (Fig. 10g). Notably, the medium dose of DEHP (200 mg/kg) exhibited lower caspase-3 expression compared to the highest dose (400 mg/kg) (Fig. 10f). Statistical analysis demonstrated a significant increase in OD in all treated groups compared to control, with the most pronounced increase observed in the higher dose groups of both compounds (*p* < 0.0001; F_6,63_ = 15.40; ƞp^2^ = 0.59; Fig. 10h).

##### Nuclear factor kappa (NF-κB)

**Fig. 11:**
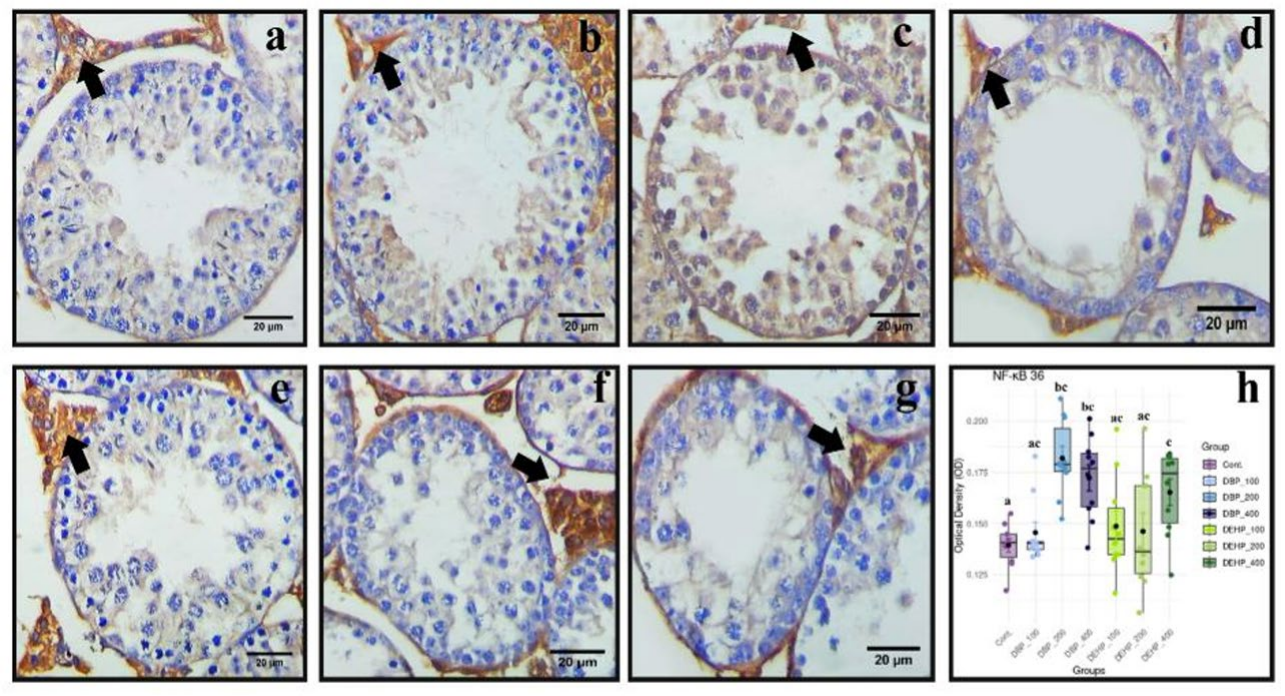
Immunohistochemical detection of NF-κB in testicular tissue at age of 36 days. Representative micrographs: (**a**) Control, (**b**) DBP 100 mg/kg, (**c**) DBP 200 mg/kg, (**d**) DBP 400 mg/kg, (**e**) DEHP 100 mg/kg, (**f**) DEHP 200 mg/kg, (**g**) DEHP 400 mg/kg. Leydig cells (thick arrow). ×400. (**h**) Optical density (OD) measurements. Different letters denote significant differences between groups (*P* < 0.05), whereas groups sharing the same letter are insignificantly different.

NF-κB expression was not detected within the seminiferous tubules in either control or most treated groups, except for the medium dose of DBP (200 mg/kg), where positive immunoreactivity was observed (Fig. 11c). However, NF-κB expression was detected in Leydig cells in both control and treated groups, showing weak staining in control (Fig. 11a) and progressively stronger intensity in the treated groups (Fig. 11b-g), particularly at higher doses for both chemicals (Fig. 11d, f & g). Statistical analysis demonstrated an insignificant increase in NF-κB optical density in most treated groups compared to control, whereas the medium dose of DBP showed a statistically significant elevation (*p* < 0.0001; F₆,₆₃ = 6.166; ηp² = 0.37; Fig. 11h).

#### At 70 days of age

##### Caspase-3

**Fig. 12:**
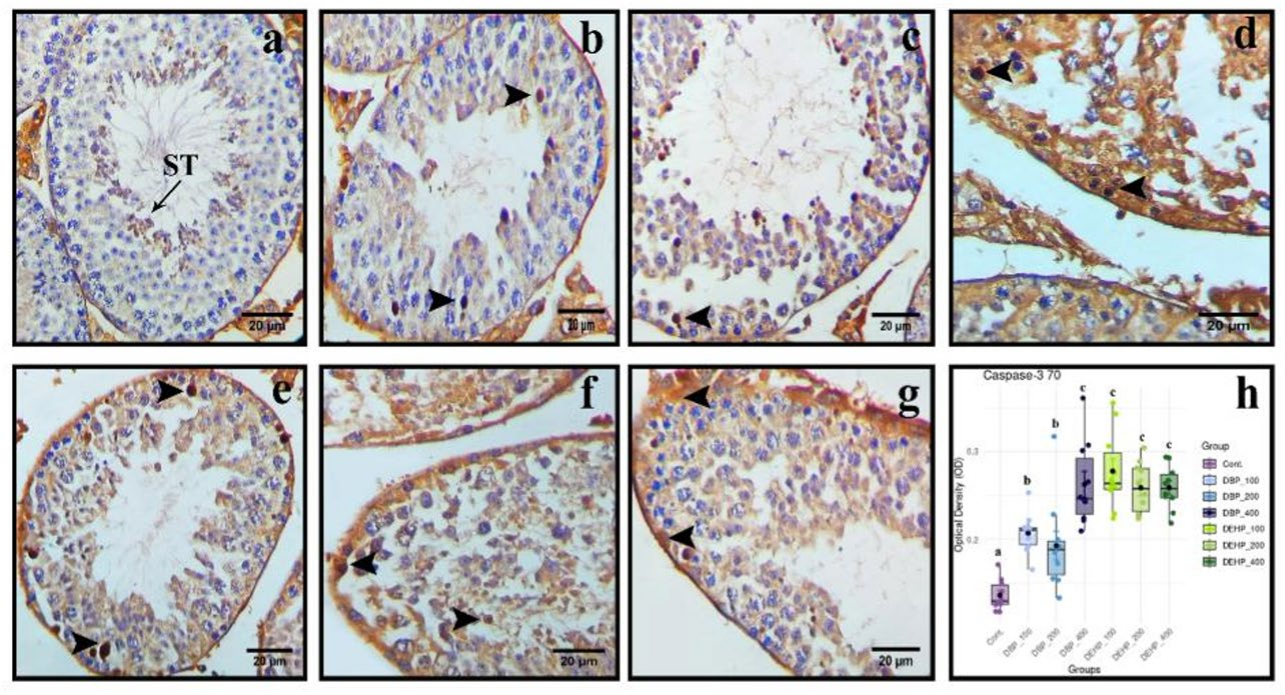
Immunohistochemical detection of caspase-3 in testicular tissue at age of 70 days. Representative micrographs: (**a**) Control, (**b**) DBP 100 mg/kg, (**c**) DBP 200 mg/kg, (**d**) DBP 400 mg/kg, (**e**) DEHP 100 mg/kg, (**f**) DEHP 200 mg/kg, (**g**) DEHP 400 mg/kg. Germ cells (arrowheads) & spermatids (arrows, ST). ×400. (**h**) Optical density (OD) measurements. Different letters denote significant differences between groups (*P* < 0.05), whereas groups sharing the same letter are insignificantly different.

Caspase-3 expression in the control group was observed only in the cytoplasm of spermatids within the seminiferous tubules (Fig. 12a). In all treated groups, expression was detected in the nuclei of some germ cells (Fig. 12b-f), with the strongest staining observed in the highest dose of DBP (400 mg/kg) (Fig. 12d) and the high doses of DEHP (200 & 400 mg/kg) (Fig. 12f & g). Statistical analysis revealed a significant increase in OD of all treated groups, with DEHP treated groups showing significantly higher levels than DBP treated groups; moreover, the highest dose of DBP exhibited a highly significant increase compared to the lower DBP doses (*p* < 0.0001; F_6,63_ = 20.10; ƞp^2^ = 0.66; Fig. 12h).

##### Nuclear factor kappa (NF-κB)

**Fig. 13:**
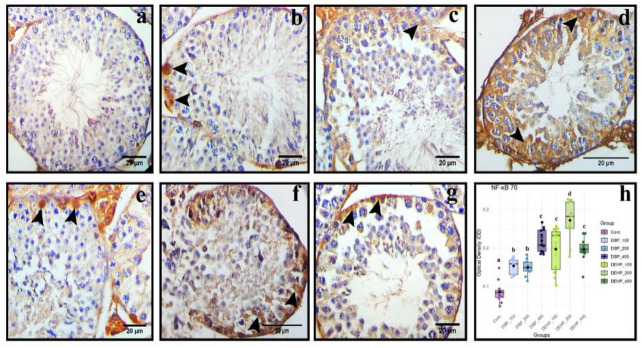
Immunohistochemical detection of NF-κB in testicular tissue at age of 70 days. Representative micrographs: (**a**) Control, (**b**) DBP 100 mg/kg, (**c**) DBP 200 mg/kg, (**d**) DBP 400 mg/kg, (**e**) DEHP 100 mg/kg, (**f**) DEHP 200 mg/kg, (**g**) DEHP 400 mg/kg. Germ cells (arrowheads). ×400. (**h**) Optical density (OD) measurements. Different letters denote significant differences between groups (*P* < 0.05), whereas groups sharing the same letter are insignificantly different.

NF-κB expression was absent in the control group (Fig. 13a). In the treated groups, germ cells in the low dose groups of both chemicals showed only nuclear stainability (Fig. 13b & e), whereas the high dose groups exhibited both nuclear and cytoplasmic stainability (Fig. 13c, d, f & g). Statistically, a significant increase in NF-κB optical density was observed in all treated groups compared to control, with the DEHP treated groups and the highest dose of DBP showing the greatest levels (*p* < 0.0001; F₆,₆₃ = 23.68; ηp² = 0.69; Fig. 13h).

## Discussion

Exposure to DBP and DEHP induced significant testicular alterations across biochemical, histopathological, and molecular markers. GSH levels were reduced, while NO and LPO levels increased, indicating oxidative stress. Histologically, treated groups showed germ cell loss, disorganization, and vacuolization, with partial recovery in lower-dose groups over time. Caspase-3 and NF-κB expression revealed activation of apoptotic and inflammatory pathways, particularly at higher doses. These findings highlight a dose dependent testicular toxicity of DBP and DEHP, mediated by oxidative stress, structural damage, and inflammation.

Postnatal exposure to phthalates in immature males has been reported to induce long-term adverse effects on reproductive development when a critical exposure threshold is exceeded, potentially limiting physiological recovery^16^. Mechanistically, this has been linked to disruption of immature Leydig cell maturation^17^and stable alterations in DNA methylation patterns, suggesting a role for epigenetic mechanisms in persistent reproductive effects^18^. Consistently, the present findings indicate a clear dose-dependent pattern. This is quantitatively supported by morphometric data at 36 days, where the percentage of damaged seminiferous tubules reached 100% in the highest dose group (400 mg/kg) for both phthalates, accompanied by a marked reduction in Johnsen’s scores to 3.5 ± 0.17. Notably, temporal evaluation indicated that while lower-dose groups showed substantial recovery of testicular architecture by day 70, high-dose groups exhibited persistent long-term impairment, as evidenced by sustained tubular damage (up to 47.0% for DBP and 58.8% for DEHP) and consistently reduced Johnsen’s scores. This sustained toxicity is associated with progressive oxidative imbalance^19^, as evidenced by depleted GSH and elevated NO and LPO levels. This oxidative overload likely contributes to enhanced apoptotic and inflammatory signaling^19,20^, as reflected by increased Caspase-3 and NF-κB expression.

In the present study, GSH depletion reflected a compromised antioxidant defense system, increasing testicular susceptibility to ROS-mediated damage. Given the central role of glutathione in maintaining cellular redox homeostasis and reproductive function^21^, its reduction likely contributed to impaired spermatogenesis and the observed histopathological alterations. Previous studies have reported reduced antioxidant levels in the seminal plasma of infertile men^22^ and decreased sperm quality associated with diminished glutathione content and trace element deficiency^23^. Additionally, glutathione deficiency has been associated with impaired sperm stability and motility^24^. Conversely, restoration of glutathione and glutathione-related antioxidant activity has been shown to alleviate toxicant-induced testicular damage and improve spermatogenic function^25,26^. It is plausible that phthalate induced depletion of endogenous glutathione played a role in the observed histopathological alterations. These alterations were accompanied by evident deterioration of spermatogenesis in the examined sections.

In the present study, elevated NO levels suggested the involvement of NO-mediated oxidative pathways contributing to testicular pathology. While physiological NO participates in sperm functions such as capacitation^28^ and acrosome reaction^29^, excessive NO negatively affects sperm motility^30^ and viability^31^ and promotes oxidative stress-induced spermatogenic arrest and apoptosis through iNOS activation^32,33^. Moreover, elevated iNOS/NO signaling has been associated with NF-κB activation^34^, leading to the release of pro-inflammatory cytokines including IL-1β, IL-6, TNF-α, and IFNs^35^, while increased NF-κB activity is a hallmark of oxidative stress-associated inflammatory testicular injury^36^. Consistent with this, increased NO and NOS levels have been recognized as biomarkers of testicular damage and male infertility^37^. Excess NO may also react with superoxide to generate peroxynitrite, enhancing lipid peroxidation and further compromising sperm membrane integrity, fluidity, motility, and fertilization capacity^32,33^.

In the present study, lipid peroxidation was markedly elevated in both DBP- and DEHP-treated groups, with DEHP inducing a more pronounced and persistent effect, suggesting greater membrane toxicity. Excessive ROS production disrupts sperm function through enhanced lipid peroxidation, mitochondrial dysfunction, and DNA damage^38^. Testicular tissue is particularly vulnerable to oxidative injury due to the abundance of polyunsaturated fatty acids in sperm membranes^39^. Lipid peroxidation reduces membrane fluidity and increases permeability, while lipid aldehydes and oxidative modifications impair proteins involved in sperm motility regulation and axonemal function^40,41^. These alterations contribute to reduced sperm motility, abnormal morphology, and impaired fertility potential^42^. Elevated MDA and other lipid peroxidation products are established indicators of oxidative damage in the testis. They have been associated with testicular cell disintegration, impaired steroidogenesis, and disrupted spermatogenesis^34^. It is suggested that phthalate exposure disrupts testicular redox balance, as evidenced by increased lipid peroxidation, elevated nitric oxide levels, and depleted glutathione. These disruptions compromise spermatogenesis and antioxidant defenses, while also promoting NO mediated inflammatory responses.

Histopathological examination of seminiferous tubules in the treated groups revealed profound structural alterations, including increased thickness of the tunica albuginea. Prominent apoptotic germ cells were observed, accompanied by extensive vacuolization and the sloughing of germ cells into the tubular lumen, reflecting severe degeneration of the germinal epithelium. The structural thickening of the tunica albuginea observed in the present study may result from a fibrotic response triggered by chronic tissue injury and localized inflammation. Fibrosis is characterized by fibroblast activation and proliferation, accompanied by excessive deposition of extracellular matrix proteins, particularly collagen, in response to pro-fibrotic mediators such as TGF-β1 released during inflammatory processes^43,44^. Moreover, repetitive microtrauma or sustained insult has been associated with aberrant healing and plaque formation within the tunica albuginea, as described in penile fibrotic disorders^45^. Collectively, these mechanisms suggest that fibrotic remodeling may underlie the increased tunica albuginea thickness observed in this study. Supporting this interpretation, immunohistochemical analysis revealed pronounced upregulation of NF-κB in Sertoli cells. Together, these findings indicate that activation of inflammatory signaling pathways within Sertoli cells likely contributes to extracellular matrix accumulation and the fibrotic changes in the tunica albuginea.

The histopathological findings, further supported by subsequent caspase and LPO analyses, provide compelling evidence of the cytotoxic impact and disrupted integrity of the seminiferous tubules following DEHP/DBP exposure. The observed vacuolization and germ cells sloughing could be a direct consequence of escalated LPO, which preferentially attacks PUFAs in cellular membranes. Spermatozoa, being particularly rich in PUFAs, are highly susceptible to LPO, resulting in membrane damage, reduced motility, and impaired fertilizing capacity^46^. The walls of the seminiferous tubules are lined by primitive germ cells and Sertoli cells, which together establish the structural and functional integrity of the seminiferous epithelium. Sertoli cells form the seminiferous epithelium barrier through tight junctions, adherens junctions, and gap junctions, thereby dividing the tubule into a basal compartment exposed to blood and lymph and an adluminal compartment that is immunologically and chemically isolated. Specialized junctional complexes between Sertoli cells, anchored to the actin cytoskeleton, form the blood testes barrier. This barrier enables Sertoli cells to regulate the adluminal microenvironment needed for germ cell development and limits the passage of cytotoxic agents into the seminiferous tubules^47^. Such seminiferous epithelium sloughing has been observed in response to toxic agents known to disrupt the Sertoli cell cytoskeleton, where loss of microtubule support leads to detachment of germ cells from the epithelium, as recently reviewed by Corpuz-Hilsabeck & Culty^48^. This further supports the interpretation that similar cytoskeletal mechanisms may underlie the histopathological changes observed in the current study, and exert their toxic effects through direct binding to β-tubulin, thereby inhibiting microtubule polymerization and disrupting microtubule dependent transport and structural support within Sertoli cells^49,50^. In the present context, the observed detachment and sloughing of the seminiferous germ cells into the tubule lumen suggest that phthalate exposure may similarly target the Sertoli cell microtubule network. Such microtubule destabilization would be expected to impair cytoskeletal integrity and junctional maintenance, ultimately leading to loss of Sertoli–germ cell adhesion and germ cells sloughing.

Seminiferous epithelium vacuolization is a common histopathological feature of Sertoli cell injury following exposure to various testicular toxicants. Defined as large basal structures (≥ 16 μm), these vacuoles correspond ultrastructurally to distended regions of the Sertoli cell smooth endoplasmic reticulum (SER), indicative of early cellular stress^51^. Accumulating evidence suggests that impairment of the Sertoli cell microtubule network plays a central role in vacuole formation, as vacuolization has been reported following exposure to microtubule targeting toxicants such as 2,5-hexanedione and carbendazim, as well as after genetic deletion of microtubule associated proteins including MAP7 and KATNAL1^52,53^. Recent studies have also shown that exposure to cytoskeleton disrupting toxicants leads to Sertoli cell structural damage and vacuolization through alterations in cytoskeleton related pathways (e.g., AKT/mTOR signaling), further supporting this mechanistic link^54^. Disruption of microtubule dependent transport is proposed to interfere with endoplasmic reticulum Golgi dynamics, leading to SER dilation and vacuole formation. Although the precise mechanism remains incompletely understood, seminiferous epithelium vacuolization is widely regarded as an early morphological marker of Sertoli cell toxicity and is frequently associated with additional lesions such as spermatid retention and germ cell sloughing^51,53^. The present study suggests that phthalate exposure may disrupt the Sertoli cell cytoskeleton and intracellular trafficking, leading to dilation of the SER and the formation of large basal vacuoles observed in the seminiferous epithelium.

Functionally, Johnsen’s scoring confirmed impaired spermatogenesis, providing a quantitative assessment of spermatogenic progression. The reduction from high control values (~ 9.9) to lower scores during the early postnatal stages (36–45 days) confirms the histopathological findings of germ cell loss, detachment, and disorganization. Notably, the partial increase in Johnsen’s scores observed at day 70 in lower exposure groups suggests a degree of time-dependent recovery or tissue remodeling following mild injury. In contrast, persistently reduced scores at higher doses indicate sustained impairment of the spermatogenic process, reflecting dose-dependent disruption of testicular architecture and function.

Furthermore, the immunohistochemical findings offer molecular evidence supporting these structural alterations. Current findings revealed that caspase-3 activation was pronounced in the nuclei of germ cells at 36 days, particularly at high exposure doses, and this elevated activity persisted until 70 days. Such prominent caspase-3 activation indicates apoptotic cell loss, consistent with the established role of caspase-3 as an apoptotic marker in germ cells^55^. The weak caspase-3 immunoreactivity observed in control spermatids may indicate a basal level of caspase activation involved in physiological germ cell turnover and remodeling during spermatogenesis, which is essential for maintaining testicular homeostasis and proper spermiogenesis as previously suggested^56,57^. While Caspase-3 upregulation indicates apoptotic involvement, confirmation using additional apoptotic markers would further strengthen these findings. The concurrent increase in testicular lipid peroxidation indicates oxidative stress-mediated cellular damage, and these early apoptotic events may promote the release of damage-associated molecular patterns (DAMPs) and other stress signals that contribute to subsequent inflammatory responses^58^. Phthalate metabolites have been reported to increase ROS generation in testicular cells, further exacerbating oxidative stress and cellular injury^59^. Consistent with these events, NF-κB activation was minimal during early stages but markedly elevated at later stages, suggesting that the delayed inflammatory response may be related to cumulative oxidative stress and DAMPs signaling. This pattern aligns with previous reports showing that DAMPs released from injured or dying cells can trigger NF-κB activation and nuclear translocation^58^, while full activation of NF-κB requires cumulative upstream signals^60,61^. Collectively, these findings suggest that phthalate-induced testicular toxicity is associated with interconnected oxidative stress, apoptosis, and inflammation, leading to progressive impairment of the seminiferous epithelium.

### Study limitations

A limitation of the present study is the absence of sperm parameter analysis, including sperm count, motility, morphology, and viability, which are important indicators of male reproductive function. These parameters were evaluated in a separate complementary study currently under review. In addition, apoptosis was assessed using Caspase-3 expression as a single marker. Therefore, the evaluation of additional apoptotic markers would have strengthened the interpretation of the apoptotic response.

## Conclusion

DBP and DEHP exposure was associated with dose- and age-dependent testicular toxicity, which may involve oxidative stress, apoptotic activity, and inflammatory responses. The observed depletion of glutathione, increased nitric oxide levels, and lipid peroxidation suggest the presence of oxidative imbalance that could contribute to germ cell damage. Histopathological alterations, including seminiferous tubule disorganization, vacuolization, and fibrosis of the tunica albuginea, indicate structural disruption of testicular architecture. In addition, increased caspase-3 expression and NF-κB activation suggest the involvement of apoptotic and inflammatory pathways in the observed testicular injury. Collectively, these findings provide evidence that phthalates may contribute to impaired spermatogenesis through oxidative stress–related cellular and structural alterations, although further mechanistic studies are required to confirm the precise molecular pathways involved.

## Materials & methods

The current study was achieved in accordance with the Egyptian laws and university guidelines for animal care. The National Ethical Committee of Assiut University, Faculty of Science Research Ethics Committee (FSREC), Egypt, has approved all the procedures in the present work with the approval number: 01–2025-0017. All procedures involving animals were conducted in accordance with the ARRIVE 2.0 guidelines.

### Chemicals

DEHP and DBP (98% purity) were purchased from Sisco Research Laboratories Pvt. Ltd. (SRL), Mumbai, India. The chemicals were merged into corn oil (used as a vehicle) before administration and were stored at room temperature.

### Experimental animals

A total of 96 immature male albino mice (Swiss strain), aged 21 days and weighing 8–12 g on average, were obtained from animal house of Faculty of Science, Al Azhar University, Assiut. The suitable temperature of 23 ± 2 °C and a lighting cycle of 12 h light/dark were taken into consideration for accommodation. Food and water were served *ad libitum*. The mice were divided into eight groups: a control group, a corn oil group (vehicle), the remaining six groups were subdivided into three groups for each of DEHP and DBP. These groups were administered by oral gavage (intragastric tube) once daily for fifteen consecutive days. Each animal received 100 µl of corn oil containing the three doses (100, 200, and 400 mg/kg bw). Twelve mice from each group were euthanized by cervical dislocation at four developmental time points (36, 45, 50, and 70 days of age; *n* = 3 per time point). No anesthesia was administered prior to euthanasia. All experimental procedures were conducted in accordance with institutional and international guidelines for the care and use of laboratory animals.

### Dose selection

Selected doses for DBP and DEHP were based on previous rodent studies. For DBP, these doses induced measurable reproductive and histopathological effects without overt toxicity^62^. For DEHP, the doses are below LD₅₀ but sufficient to produce dose dependent reproductive effects in mice^63^.

### Biochemical investigations

Biochemical studies were carried out on ages 36, 45, 50 and 70 days. Testicular tissues were carefully excised, washed in cold saline, and stored at −80 °C until biochemical assessments. Specimens were prepared as 10% weight/volume homogenate using homogenizer (Unidrive 1000 D, Germany). Phosphate buffer solution (PBS) was used and pH was adjusted at 7.4. The homogenates were centrifuged at 5000 rpm for 20 min and the supernatants were kept frozen at −20 °C for the subsequent biochemical assays. Levels of nitric oxide (NO), reduced glutathione (GSH), and lipid peroxidation (LPO) were measured in the supernatant of testicular tissue homogenates as indicators of oxidative stress and antioxidant status using standard colorimetric methods as previously described^64–66^.

### Histopathological studies

Histological studies were carried out on ages 36, 45, 50 and 70 days. Testes were dehydrated in a series of ethanol, cleared in cedar wood oil, washed in chloroform, embedded in paraffin wax and transverse serial sections were prepared at 4 microns thick. The staining procedures were applied according to Drury & Wallington^67^ as follows: Harris haematoxylin and eosin for general histological studies. Masson’s trichrome stain to clarify collagen fibers^68^. Photomicrographs were acquired at known magnifications using a microscope-mounted digital imaging system. Spermatogenesis was evaluated using the Johnsen scoring system (1–10 scale) as described by Johnsen^69^. In addition, the percentage of damaged seminiferous tubules was determined by counting tubules exhibiting histopathological alterations such as germ cell detachment, disorganization of the seminiferous epithelium, and cytoplasmic vacuolization, relative to the total number of examined tubules in each section, as a quantitative measure of testicular injury, using the following equation:

Damaged seminiferous tubules (%) =$\:\:\frac{\mathrm{N}\mathrm{u}\mathrm{m}\mathrm{b}\mathrm{e}\mathrm{r}\:\mathrm{o}\mathrm{f}\:\mathrm{d}\mathrm{a}\mathrm{m}\mathrm{a}\mathrm{g}\mathrm{e}\mathrm{d}\:\mathrm{t}\mathrm{u}\mathrm{b}\mathrm{u}\mathrm{l}\mathrm{e}\mathrm{s}}{\mathrm{T}\mathrm{o}\mathrm{t}\mathrm{a}\mathrm{l}\:\mathrm{n}\mathrm{u}\mathrm{m}\mathrm{b}\mathrm{e}\mathrm{r}\:\mathrm{o}\mathrm{f}\:\mathrm{e}\mathrm{x}\mathrm{a}\mathrm{m}\mathrm{i}\mathrm{n}\mathrm{e}\mathrm{d}\:\mathrm{t}\mathrm{u}\mathrm{b}\mathrm{u}\mathrm{l}\mathrm{e}\mathrm{s}}\times\:100$.

### Immunohistochemical investigation

The immunohistochemical investigation was carried out on formalin fixed testes collected at ages of 36 and 70 days. The specimens were processed, embedded in paraffin, and the tissue sections were cut at 4 μm. Sections were subjected to antigen retrieval using Tris–EDTA buffer (pH 9.0) at 90 °C for 20 min. The block of endogenous peroxidase was done by incubating tissue sections in 3% H_2_O_2_, followed by preincubation overnight at 4 °C in 1% bovine serum albumin in PBS. The sections were stained for 30 min at 37 °C, using the following antibodies that showed reactivity in mice species: a rabbit polyclonal anti-NF-κB (1:200; ABclonal Technology, Wuhan, China; Cat. No. A19653), a rabbit monoclonal anti-caspase-3 (1:200; ABclonal Technology, Wuhan, China; Cat. No. A11953) according to the method described previously^70^. Sections were counterstained with haematoxylin, and analyzed using a digital imaging system. In parallel, tissue specimens, in whom the primary antibodies were omitted and replaced with buffer, served as negative controls.

### Statistical analysis

Data were expressed as mean ± SEM. Statistical analysis was performed using one-way ANOVA followed by Tukey’s multiple comparison test as a post hoc test. These analyses were carried out using Prism software for windows, version 5.0 (Graph pad software Inc., San Diego, California, USA) and Excel (Microsoft office 10). Analysis of optical density (OD) was carried out using the software Image J (the JAVA SE Runtime Enviroment, version 6).

## Data Availability

Correspondence and requests for materials should be addressed to Heba E. Aboulqasem.
